# Heat Flux Sensing for Machine-Learning-Based Personal Thermal Comfort Modeling

**DOI:** 10.3390/s19173691

**Published:** 2019-08-25

**Authors:** Wooyoung Jung, Farrokh Jazizadeh, Thomas E. Diller

**Affiliations:** 1 Department of Civil and Environmental Engineering, Virginia Tech, Blacksburg, VA 24061, USA; 2 Department of Mechanical Engineering, Virginia Tech, Blacksburg, VA 24061, USA

**Keywords:** HVAC, comfort-driven operation, thermal comfort, heat flux sensors, thermoregulation mechanism, personalized thermal comfort models

## Abstract

In recent years, physiological features have gained more attention in developing models of personal thermal comfort for improved and accurate adaptive operation of Human-In-The-Loop (HITL) Heating, Ventilation, and Air-Conditioning (HVAC) systems. Pursuing the identification of effective physiological sensing systems for enhancing flexibility of human-centered and distributed control, using machine learning algorithms, we have investigated how heat flux sensing could improve personal thermal comfort inference under transient ambient conditions. We have explored the variations of heat exchange rates of facial and wrist skin. These areas are often exposed in indoor environments and contribute to the thermoregulation mechanism through skin heat exchange, which we have coupled with variations of skin and ambient temperatures for inference of personal thermal preferences. Adopting an experimental and data analysis methodology, we have evaluated the modeling of personal thermal preference of 18 human subjects for well-known classifiers using different scenarios of learning. The experimental measurements have revealed the differences in personal thermal preferences and how they are reflected in physiological variables. Further, we have shown that heat exchange rates have high potential in improving the performance of personal inference models even compared to the use of skin temperature.

## 1. Introduction

### 1.1. Emergence of a Personalized Operational Paradigm in HVAC Systems

Human-centered operation of Heating, Ventilation, and Air-Conditioning (HVAC) systems has gained attention in the last decade given its potentials for improved energy efficiency by enhancing occupant comfort and reducing energy consumption [1,2,3]. Conventionally, in the design and operation of HVAC systems, collective models, most notably the Predicted Mean Vote (PMV) model, have been used to identify the configuration of the building systems according to the occupant comfort and to reflect occupants’ perspectives. The PMV model has been standardized by American Society of Heat, Refrigerating, and Air-conditioning Engineers (ASHRAE) [4] to render indoor environments thermally acceptable for majority of occupants by employing two human-related parameters of clothing level and metabolic rate. However, it has been shown that actual comfort zones often deviate from those obtained through the PMV model. The PMV model presumes that the neutral state on the ASHARE thermal sensation scale is the preferred state by occupants [4]. However, it has been demonstrated that occupants desire diverse thermal sensations from lower to higher temperatures as their comfort state at an individual level [5]. More importantly, the generalization of human-related parameters in the PMV model according to standardized experimentation has played a part in the observed discrepancies [6]. The generalization of met unit for metabolic rate stemmed from controlled experiments on selected samples [7], which may not be universally applicable. Clothing insulation is also commonly considered to follow standard values, which might not reflect the real experience of an individual. These mismatches have resulted in occupants’ thermal discomfort and low energy efficiency [8].

On the other hand, lack of human-building interaction has compounded the suboptimal performance of current HVAC systems. Thermostats, commonly used interfaces for user input, reflect ambient condition variations at one point of a thermal zone (a collection of rooms or subspaces that are supplied by the same air supply unit). They might be improperly located and users might be hesitant to adjust the thermostats due to the authority concerns in non-residential environments [9,10]. Furthermore, in non-residential units, generalized temperature setpoints are set by facility managers regardless of actual users’ perspective [11]. Even in case of user interaction, using temperature setpoints at the thermostat location shifts the focus from individual user comfort perception to a one-point spatial temperature representation. These limitations, associated with the use of thermostats, have motivated the research on personal means of comfort measurement and modeling for HVAC operations.

In past decade, research efforts have explored the potentials of personalization techniques in the control of HVAC systems [12,13]. In other words, research has shifted towards Human-In-The-Loop (HITL) control strategies [14]. The prevalence of Internet of Things (IoT) and connected devices has paved the way for such developments to move from either use of thermostats or conventional hard-copy questionnaires for occasional collection of occupant feedback. Therefore, electronic mediums (e.g., web-based [11] and smartphone-based [15]) have been proposed to facilitate data collection processes and improve the flow of information from occupants to building systems, enabling occupants to provide their feedback for the control of HVAC systems. These efforts further have proceeded with compiling data on personal thermal feedback (using different thermal sensation scales) in concomitant with measuring the environmental ambient and contextual conditions (such as temperature, humidity, clothing level, and seasonal information) in the form of comfort datasets [3,16,17,18]. Leveraging such datasets, machine learning (ML) and statistical modeling techniques have been used for creating personalized thermal comfort profiles mapping the ambient conditions to the probability of personal thermal satisfaction and showing the personal sensitivities to different ranges of temperature variations (i.e., thermal comfort sensitivity) [3,16,19,20]. These profiles have been further integrated into the operation of HVAC systems for comfort-driven HVAC operations [1,2,16,18,20].

Studies have shown that challenges could emerge when using direct user feedback and its association with ambient thermal conditions for occupant thermal comfort inference. The studies, which have relied on the survey-based methods and thermal ambient conditions for personal comfort modeling, have demonstrated moderate accuracies (60–75%) [17,21,22]. In an example study, Kim et al. [21] have reported the median accuracy of 73% by using 23 contextual (and non-physiological; features including thermal preference, clothing insulation, control characteristics, occupancy status, historical occupancy frequency, time features, indoor and outdoor temperature and humidity features, sky cover, precipitation, indoor airflow, and HVAC features like damper position, heating output, and discharge air temperature) for training their ML algorithms. This is, to the best of our knowledge, the largest number of features used in thermal comfort inference studies. Among these attributes are human-related parameters that have been self-reported by users. In order to address the challenges, associated to human subjective feedback, research efforts have investigated the potential of using additional human-related features (such as the human body physiological responses) as inputs to ML models of thermal comfort inference. Recent studies have reported improved performances of comfort inference, up to 85% median accuracy, by using the human physiological response data [23]. We have presented a detailed and quantitative review on HITL HVAC operations using personal thermal comfort models in [14]. The most representative human body response with respect to ambient environment is the regulation of blood flow to skin. Blood vessels get dilated or constricted (i.e., vasodilation and vasoconstriction, respectively) to regulate heat dissipation through the skin and preserve the core body temperature [7]. Therefore, skin temperature variations have been shown in previous studies to have high correlation with reported users’ thermal sensations [24,25]. Specifically, the infrared imaging technology has drawn attention due to its non-intrusive nature [23,26,27], which is one of the important factors in feasibility of human-building interaction applications [28,29,30,31].

### 1.2. Ubiquitous Thermophysiological Sensing

Our vision is to move towards applicable sensing technologies [28,29,30,32,33] that enable the measurement of human thermophysiological responses, which can bring about flexibility for comfort-driven HVAC system operation. In previous research efforts, skin temperature has been predominantly used as an important attribute either by using the infrared imaging technology [23,26] or temperature sensors in wearable forms [17,18], which put the focus on a single thermoregulation feature. Pursuing the objectives of ubiquity, applicability, and non-intrusiveness, in recent years, we have proposed and investigated novel physiological sensing techniques using RGB cameras (by indirect measurement of blood perfusion to skin surface) [30,34] and Doppler radar sensors (by measuring the variations in respiration patterns) [28,32,33]. In this study, as another modality of a sensing system at the intersection of the aforementioned criteria, we have sought to demonstrate the potentials of heat flux sensors for human-building interaction in thermal comfort management. To this end, our research questions were as follows:

(1) How could heat flux sensors be used for improved personal comfort inference and comfort-driven HVAC operation?
(2) What is the relationship between skin temperature and heat exchange rate?
(3) Which feature between skin temperature and heat flux could perform better in personalized comfort inference?

A heat flux sensor is capable of measuring heat exchange rates between two objects. Accordingly, it can directly quantify heat exchange rates between the human body and the ambient environment. Given the human thermoregulation mechanism, which adjusts the body heat dissipation, we have hypothesized that variations of heat exchange rates between the human body and ambient environment are associated with changes of human thermal perceptions, which are varied at an individual level (the research question #1). By measuring the variations of most commonly used thermophysiological attribute, skin temperature, in parallel with heat exchange rates we have explored the relationship between human thermoregulation mechanism and human-environment heat exchange (the research question #2). Lastly, we evaluated the use of heat exchange rate as attribute for personal comfort inference. By comparing this feature with skin temperature, the potential of utilizing it as a single input has been assessed (the research question #3). Given that this new feature could provide a more explicit parameter of human thermoregulation, we have hypothesized that the performance of personal comfort inference could be improved. To address the research questions, we have conducted two experimental studies with 32 human subjects (19 males and 13 females). We used transient temperature setups from low (around 20 °C) to high (around 30 °C) and high to low in a thermal chamber and measured the variations of heat exchange rates and skin temperatures of facial and wrist areas that are often exposed in indoor environments and have commonly been the subjects of measurements in similar studies [24,35,36]. Such areas have been preferred, when it comes to thermoregulation state quantification due to their direct indications compared to the areas covered by clothes.

The rest of the paper is structured as follows: Section 2 explains the thermal interaction mechanism between human body and environment and the human thermoregulation mechanism, which we have leveraged in this study. In Section 3, we introduce a heat flux sensor used in this study, explain experimental procedures, and present signal processing methods that we utilized for noise reduction and supervised-learning algorithms that were used for personalized thermal comfort inference. Section 4 discusses the results and Section 5 presents the conclusions of our study.

## 2. Physiological Attributes in the Thermal Comfort Modeling

Thermal comfort is a subjective satisfaction evaluation with respect to the ambient environment which involves occupants’ physical, physiological, psychological and other processes [4]. In other words, thermal comfort could vary with the same ambient environment depending on human-related variables. Therefore, as noted, the recent research efforts sought to account for occupants’ thermophysiological responses with respect to ambient temperature. In this way, the measurements could implicitly reflect the human-related variables and help identify causality. The human thermoregulation mechanism is an autonomous body response, regulated by the hypothalamus in the brain. This part of brain gathers thermal data from the skin and arterial blood, and then stimulates thermophysiological responses, required for body temperature regulation [7]. ASHRAE [7] describes the following thermophysiological responses:

- Blood flow to skin: The most widely used physiological feature which causes skin temperature variation (i.e., vasodilation and vasoconstriction),
- Sweating: A defense mechanism that cools the skin and increases heat loss from the core,
- Respiration: A way of losing sensible and latent heat, and
- Heart rate: Moderate metabolism indicator.

These variations in thermophysiological responses are converted into several modes of heat exchange (e.g., convective, evaporative, and radiative heat losses from skin, and convective and evaporative heat loss from respiration). ASHRAE [7] also states that the total metabolic rate is equal to sum of all heat exchange modes and heat storage in skin and core compartments unless there is an external work (Equation (1)):

(1) $$M-W=q_{sk}+q_{res}+S$$

where M: rate of metabolic heat production, W: rate of mechanical work accomplished, $q_{sk}$: total rate of heat loss from skin, $q_{res}$: total heat loss through respiration, and *S*: rate of heat storage in skin and core compartments. ASHRAE [7] states that 27 parameters are effective in driving the metabolic rate of human body (e.g., heat transfer coefficients, clothing area factor, etc.). The PMV model has provided a practical estimate of this variable [37] through controlled experimental studies. As noted, although this model has been used for decades [6], studies have shown incompatibility for personal thermal comfort inference due to its generalization [21,22].

Therefore, the previous studies, especially in the field of indoor thermal comfort quantification, have explored potentials of contextual measurements of physiological responses at the individual level. Choi and Loftness [24] and Yi and Choi [27] demonstrated that skin temperature variations, captured from wrist and facial areas, are associated with individual’s thermal sensation. Choi et al. [38] examined heart rate variations under different activity levels (e.g., lying, sitting, and cycling) and reported that they can be a potential indicator/parameter for human thermal comfort inference. Using subjects’ electrocardiogram, Liu et al. [39] demonstrated that the ratio of absolute power in low frequency (0.04–0.15 Hz) and high frequency (0.15–0.40 Hz) increased when human subjects were feeling thermal discomfort. These research efforts have demonstrated that contextual physiological responses could aid in personal thermal comfort inference. The value of physiological features, such as electrodermal (EDA), skin temperature, and photoplethysmography (PPG) from wearable sensors for detecting user physical and mental status have been explored in other domains as well (e.g., [40,41,42]).

Motivated by such studies, research efforts for comfort-driven HVAC control has paid attention to physiological feature analysis in recent years. Advances of wearable/non-intrusive measurement techniques have facilitated this research trend. As discussed in our previous studies [28,29], the key attribute for comfort-driven HVAC operations is feasibility of sensing techniques—they should non-intrusively quantify thermophysiological responses in a timely manner and be ubiquitously available for scalability. Under these constraints, recent studies employed wearable/non-intrusive measurement techniques for capturing users’ physiological responses and devised personalized comfort inference models [13]. Li et al. [17,18] developed a smartwatch application to collect human subjects’ thermal votes, as well as their physiological attributes (skin temperatures and heart rates) for personalized comfort inference models. Ranjan and Scott [26] employed an infrared imaging technology for measuring facial and hand skin temperatures and classified the necessity of energy use according to personal thermal comfort sensations. Sim et al. [35] assessed the use of wrist temperatures, using various wrist-type wearable sensors in the market, as input parameters to ML algorithms and showed the potentials in thermal sensation estimation. Given the diversity in space/building types and individual differences among building occupants, as noted, we have investigated an alternative modality of physiological sensing technique (i.e., heat flux sensing) for improved personal thermal comfort modeling.

## 3. Methodology

### 3.1. Distributed Sensing and Control Framework

**Figure 1:**
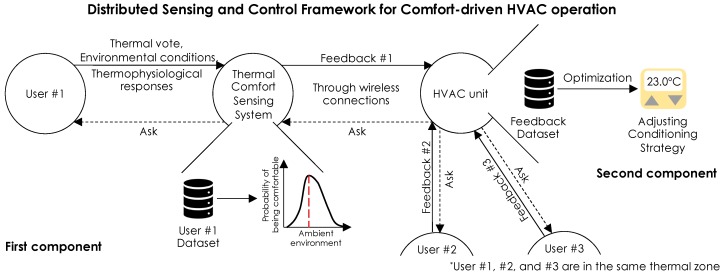
The distributed sensing and control framework for comfort-driven HVAC operation.

Figure 1 illustrates a generic framework for HITL HVAC control that leverages human sensor proxies for distributed feedback to the control logic for operational optimization. Our research efforts of integrating individuals’ thermophysiological responses into the thermal comfort sensing system—i.e., collecting users’ thermal votes and physiological responses for personal comfort modeling—contribute to the first component of the framework. By enhancing the performance of personalized thermal comfort inference or characterization, accurate feedback can be fed to the HVAC controller system, which optimizes the desired environments for multiple users through adaptive operations. Even though, depending on the optimization approach, different types of feedback might be required from the thermal comfort sensing system [43], creating accurate thermal comfort models in the first component of the framework is of critical importance for optimum performance. In the first component, each user’s thermal comfort behavior is characterized by analyzing reported thermal votes as well as physiological responses and environmental data measurements through wearable or non-intrusive sensors. The key factor is to have a feasible thermal comfort sensing system, which meets the criteria of ubiquity, non-intrusiveness, and applicability with sufficient accuracy.

The data is used to develop personal models of thermal comfort to profile individual perceptions/preferences by using stochastic modeling methods such as Bayesian network modeling [43] or ML approaches [18,21]. In the second component, the thermal comfort sensing system provides personal feedback, derived from personal thermal comfort models, as required from the HVAC system, for comfort-driven HVAC operation. Using Wi-Fi or Bluetooth protocols, the data is wirelessly communicated (processed data could be communicated with a frequency of one point with a period of one to five minutes) to a smart thermostat or a Building Energy Management (BEM) system. Using different operational strategies, the feedback could be used to control the environment either for individuals or collectively [1,2,3,43,44,45,46,47]. This study contributes to the sensing component toward enhancing feasible physiological sensing systems, which enable accurate measurement of interactions between the human body and the ambient environment. The following subsection present the steps that we followed to answer the aforementioned research questions.

### 3.2. Heat Flux Sensing

**Figure 2:**
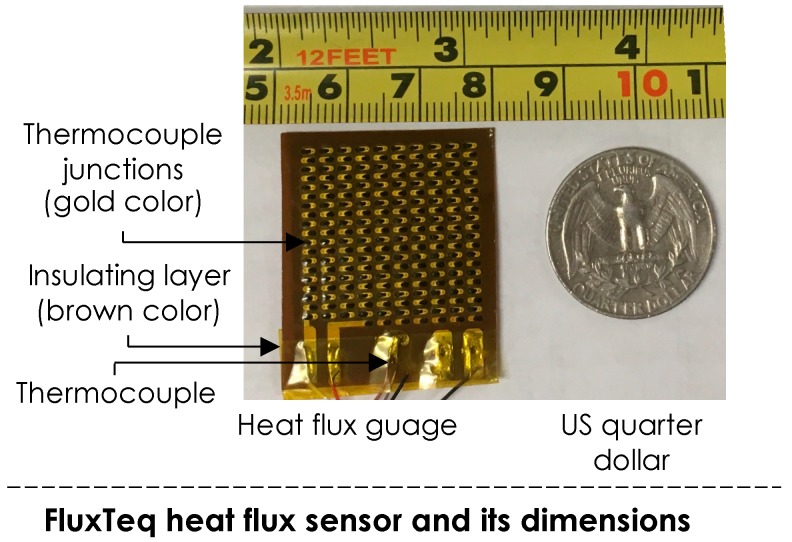
Heat flux gauge used in our study [51].

Heat flux sensors generate electrical voltage signals proportional to the heat exchange rate through the surface of the transducer. The sensor outcome is used to calculate the heat exchange rate, which is measured in W/m^2^. In this study, we have employed a heat flux gauge, as shown in Figure 2. This gage is manufactured by FluxTeq [48] and specified in American Society for Testing and Materials (ASTM) E2684–17 [49]. This device consists of a differential thermopile made through holes in a sheet of polyimide (Kapton), which is about 150 μm thick with a corresponding time response of 0.6 s. A large number of thermocouple junction pairs across the heat flux gauge generate a robust voltage signal with a reduced temperature disruption to the surface [50]. This heat flux sensor also has a thermocouple mounted on the gauge (Figure 2). Therefore, the same sensing device have been used for collecting both heat exchange rate from skin, as well as skin temperature.

The advantage of this sensing technology is its flexibility and design that can be adopted for different use cases. The transducer form enables the integration of this technology into wearable devices such as smartwatches and create a seamless communication between users and building systems. The smartwatch manufacturers are moving towards sensor integration into the smartwatch bands as well to enable data acquisition and wireless communication capabilities.

### 3.3. Experimental Procedure

Using the heat flux sensor, we conducted experimental studies upon receiving the approval of Virginia Tech’s Internal Review Board (IRB) and obtaining informed consents from all human subjects. The details of two experimental procedures are as presented in Table 1. It should be noted that we have recruited volunteer participants and did not eliminate any data to present the data from equal number of female and male participants. Therefore, the number of occupants from each gender group is different. We focused on two areas of skin: (1) facial and (2) wrist areas given that both areas are often exposed indoors and have been a common area of measurement using non-intrusive and wearable sensing technologies. Although it is not feasible to place heat flux sensors on a facial area during operation of the building, the experiment was conducted to explore the thermophysiological responses of the facial skin for applications of vision-based techniques on the facial area. As noted, the facial skin area is often used by the studies investigating its thermophysiological responses [23,26,29,30,34,52], addressing the association between the heat change rate and skin temperature in the facial area is important in HITL HVAC operations. In other words, the aim of the first experiment was to investigate the relationship among ambient temperature, skin temperature, and the heat exchange rate for such applications.

**Figure 3:**
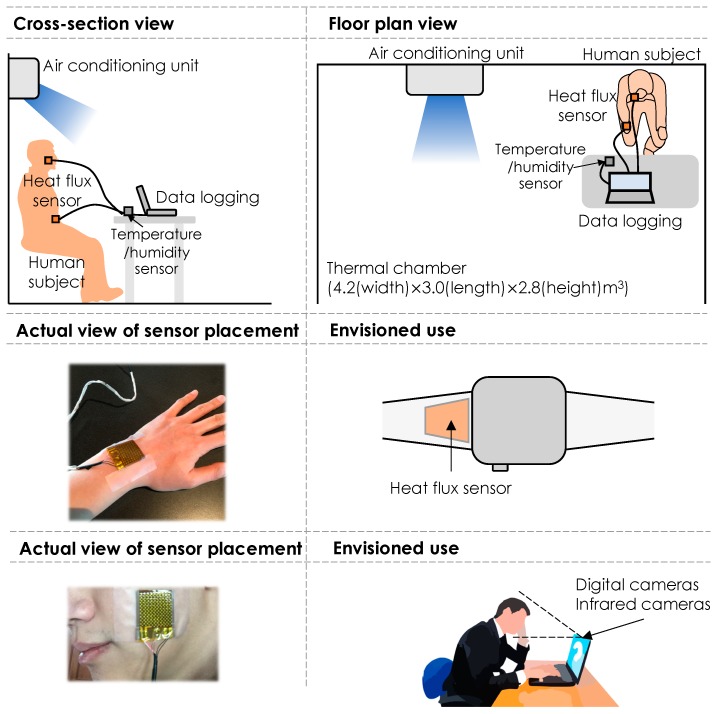
Experimental setup in a thermal chamber and the placement of the heat flux sensor in the experiment along with their potential use cases.

Figure 3 illustrates the experimental setup for this study. We utilized a test bed with dimensions of 4.2 (width) × 3.0 (length) × 2.8 (height) m^3^. The test bed is equipped with a dedicated air-conditioning unit for temperature control. Human subjects were seated in a way that they were not directly exposed to the flow of the conditioned air. The experiments were designed to have gradual temperature variations to simulate the realistic scenarios of temperature variations, in which prolonged acclimation time was not taken into account. At each experimental run, we only had a single human subject in the test bed to eliminate the other human subjects’ influence on thermal conditions and thermal preferences. With multiple subjects, a subject could be psychologically influenced by others’ thermal comfort perception/preference and be thermally impacted by presence of others.

The heat flux gauge (Figure 2) was used to measure the heat flux and skin temperature at a sampling rate of 3.0 Hz. We used a DHT-22 sensor, connected with an Arduino microprocessor, to measure the ambient temperature and humidity at 0.5 Hz. All subjects declared no cardiopulmonary-related illnesses at the times of the experiments. Thermal comfort votes were collected using a simple survey interface and a thermal preference scale with 11 degrees from −5 to 5 representing a range from preferring a cooler environment (uncomfortably warm) to preferring a warmer environment (uncomfortably cool). The adopted scale helps us measure perception, preference and the intensity associated with the thermal votes (for more details on the scale characteristics see [53]). The procedure of the experiments was as follows:

(1) Each participant waited for 10 to 15 min outside the thermal chamber in the building to ensure that their thermoregulation has reached a stabilized state. This acclimation time was considered prior to the experiment to ensure that potential activities prior to the experiment (e.g., walking from outside) do not bias the outcome of the experiment. Prior activities could affect the participant’s metabolic rate and therefore, his/her thermal sensation might be affected by other factors.
(2) The participant entered the conditioned thermal chamber (with an ambient temperature at around 20 °C) and the heat flux gauge was attached to the skin.
(3) All sensors (a heat flux, a thermocouple, and a temperature/humidity sensor) were activated and data collection was started.
(4) At the beginning of the experiment, the participant reported his/her thermal preference from a five-point thermal preference scale −5 (preference to be cooler or uncomfortably warm) to 5 (preference to be warmer or uncomfortably cool).
(5) Then, the ambient temperature varied at a pace of around 1 °C per 5 min. For the first experiment, the temperature raised until around 30 °C and for the second experiment, it increased until 30 °C and then decreased back to about 20 °C as described in Table 1.
(6) The participants were instructed to report their thermal preference every time that they feel a change in their thermal preference.

During the experiments, to avoid biases in participants’ thermal votes based on their historical information, the temperature values in the testbed were not revealed to them. They were only aware that we will change the temperature during the experiment.

### 3.4. Data Post-Processing Analysis

After data collection, we performed a number of post-processing steps on the data from heat flux, skin temperature, and ambient environment sensors to achieve higher signal-to-noise (SNR) ratios. The processed data were used for correlation analysis and comfort inference modeling, which are presented in the following subsections.

**Figure 4:**
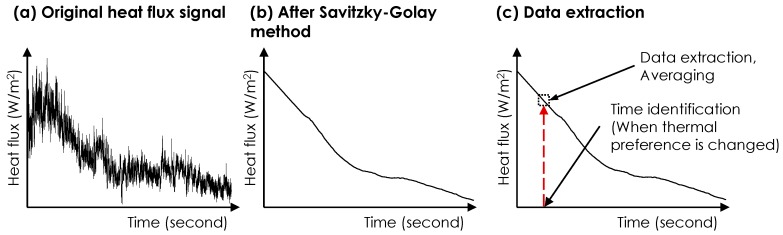
Post-processing of heat flux data.

*Heat flux and skin temperature data*: As indicated in Figure 4, we performed two post-processing steps to extract heat flux data: (1) the Savitzky-Golay filtering and (2) data sampling for feature extraction. The first step was used for noise reduction of the signal. The heat flux data include a high degree of noise due to the natural air movement in the room [54]. Benefiting from its strength in maintaining the shape and the height of the raw signal waveforms, the Savitzky-Golay filtering method was used in this study [55]. The approach utilizes the local least-squared polynomial approximations for noise removal. We used degree one polynomials and a window size of 3001 for the input parameters, which were empirically selected based on the data processing results. In the second step (i.e., feature extraction), we extracted the average heat flux data over a 30-s window associated with each thermal preference vote. Our observations indicated that participants did not change their thermal preferences for at least 30 s after each new vote (e.g., a subject reported +2 at 10:40 a.m. and he/she maintained it for at least 30 s). The sampled average values were associated with thermal preference votes. The sampled signal values between two consecutive votes were labeled with the earlier vote value. For example, if a participant voted +2 (meaning a preference for warmer environment), the sampled data were labeled with that until the time of a new vote.

**Figure 5:**
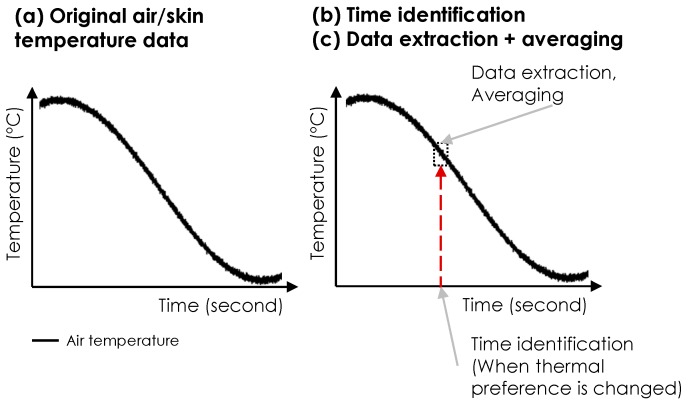
Post-processing of skin temperature and environmental data (a schematic figure).

*Environmental and skin temperature data*: The environmental data, as shown in Figure 5, has less apparent noise, compared to data captured through heat flux sensor. The data sampling for feature extraction was conducted in the same way. The average values over 30 s were used in the analyses.

### 3.5. Correlation Coefficient Analysis and Thermal Comfort Inference

*Variations/Relations of physiological responses*: As part of the analysis, we looked at the personal physiological features during the comfortable state. Utilizing the post-processed data, we identified human subjects’ physiological responses (i.e., heat flux and skin temperature), as well as environmental data during the comfortable state. Furthermore, we investigated the correlation among heat exchange rate, skin temperature, air temperature, relative humidity, and thermal preferences to answer the research questions. As all parameters are either continuous or ranking variables (only thermal preference data is a ranking variable), the Pearson correlation coefficient was used in this study (Equation (2)):

(2) $$r=\frac{\sum_{i=1}^{n}\left(x_{i}-\bar{x}\right)(y_{i}-\bar{y})}{\sqrt{\sum_{i=1}^{n}{\left(x_{i}-\bar{x}\right)}^{2}}\sqrt{\sum_{i=1}^{n}{\left(y_{i}-\bar{y}\right)}^{2}}}$$

*Thermal comfort inference*: For this part of the analysis, we only used the data from experiments that used the heat flux sensors on the wrist. In developing the personalized thermal comfort inference models using ML, we used heat flux, skin temperature, and environmental data, coupled with thermal preference votes from participants. For classification analyses, we modified the labeling to include three classes: all uncomfortably warm votes (from −5 to −1) were labeled as −1 and all uncomfortably cool votes were labeled as +1. The three-class thermal vote scale is the most widely used convention across different studies [18,21,22]). This convention is a practical approach for control purposes as it has been shown that by using a three-class labeling convention, a more accurate outcome could be obtained [14]. As noted, all the data points between two consecutive thermal votes were labeled with the earlier vote. For example, for a subject that voted no change at 1:45 p.m. and changed the vote to cooler at 1:54 p.m., the data points between these two votes were labeled as no change (0). In this way, we could increase the number of data points in developing the models and evaluating them under more diverse conditions.

We used three ML algorithms: Random Forest (RF), Support Vector Machine (SVM), and multinomial Logistic Regression (LR), which have been utilized in comfort inference modeling in previous studies that used skin temperature as one of the input parameters [17,18,26]. By using the same algorithms, we intended to demonstrate the potentials of employing heat flux data in modeling personal thermal comfort inference.

We investigated different scenarios of training and testing. The details of scenarios are presented in Table 2. As noted, we have exposed the participants to two conditions of increasing and decreasing ambient temperatures. These conditions were considered to provide an insight into training of the personal comfort models under varying operational conditions and their impact on modeling performance. In addition, we have used different combinations of features: (1) air temperature, (2) skin temperature and air temperature, (3) heat flux rates and air temperature, and (4) skin temperature, heat flux rates, and air temperature. The first combination was used as a benchmark to demonstrate the performance of participatory sensing though occupants’ voting systems—employing only environmental variables, coupled with occupants’ votes for thermal comfort inference. The remaining combinations were used to address the research questions.

For the first and second scenarios, given that train and test sets were separated, (similar to a cross validation process) we created 10 subsections of the training set and trained inference models on randomly selected groups by leaving one subsection out at each time. We then used the entire test data set for testing the general performance. For the third scenario, we performed the standard 10-fold cross validation. For all scenarios, we used the averaged performance metrics in the Results section.

## 4. Results

### 4.1. Analysis of Physiological Responses

**Figure 6:**
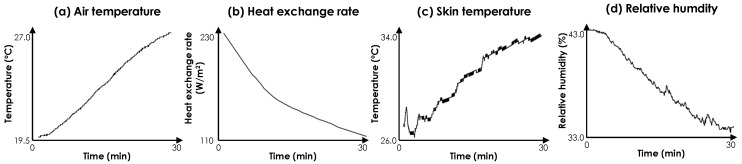
Data captured from human subject #5.

*Study of physiological variables on the facial skin*: Figure 6 shows the variations of each measured parameter for an example participant in the first experimental study—i.e., measuring the heat flux and skin temperature on the cheek. The second graph with the heat exchange rate data (Figure 6b) has been plotted after applying the Savitzky-Golay filtering. The rest are the raw ambient condition data. This example shows representative trends in our dataset. At the beginning of the experiment, with the lower ambient temperature values, a higher heat exchange rate was observed. This demonstrates that the human body dissipated more heat with low air temperatures due to the temperature gradient between the body and the environment. In contrast, the measured skin temperature was low and close to the ambient temperature in lower air temperatures. The skin temperature could be affected by the vasoconstriction mechanism (reduction of the blood flow to the surface of the skin due to thermoregulation) and the lower ambient temperature in the room. Since a high rate of heat is getting dissipated, the thermoregulation mechanism gets triggered to reduce the heat exchange rate and improve thermal comfort experience. All of the subjects reported that they preferred a warmer condition at the beginning of the experiment, reflecting a lack of comfort perception. As the temperature increased, we could observe a decreasing trend in the heat flux values and an increasing trend for the skin temperature. At the end of the experiment, all subjects expressed their preference for cooler conditions with thermal preference ratings varying between −1 and −5. The consistent decrease in relative humidity throughout the experiment was not planned in the experiments. We have conjectured that since our testbed was a closed space with no windows, minimal infiltration has resulted in such a trend for relative humidity. Therefore, we do not consider relative humidity as a factor in our analyses and we solely focused on the ambient temperature as the main driving factor in the change of thermal votes. This also corresponds to the demonstrations in the literature [22,53], which state that the major driver for thermal comfort vote is the change in air temperature.

**Figure 7:**
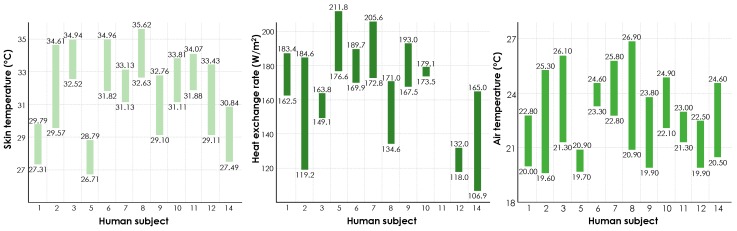
Participants’ skin temperature, heat exchange rate, and air temperature during the comfortable state.

Figure 7 presents the cheek skin temperature, heat exchange rate, and air temperature for the temperature ranges during which each human subject expressed a comfortable vote. This visualization demonstrates that different individuals perceive thermal comfort in a certain heat exchange rate and if out of this range, thermal discomfort is experienced. This also holds true for the other two variables. Moreover, it is observed that every subject had a distinct range for each attribute, which demonstrates the need for developing personalized comfort models or profiles for comfort-driven HVAC operations.

**Figure 8:**
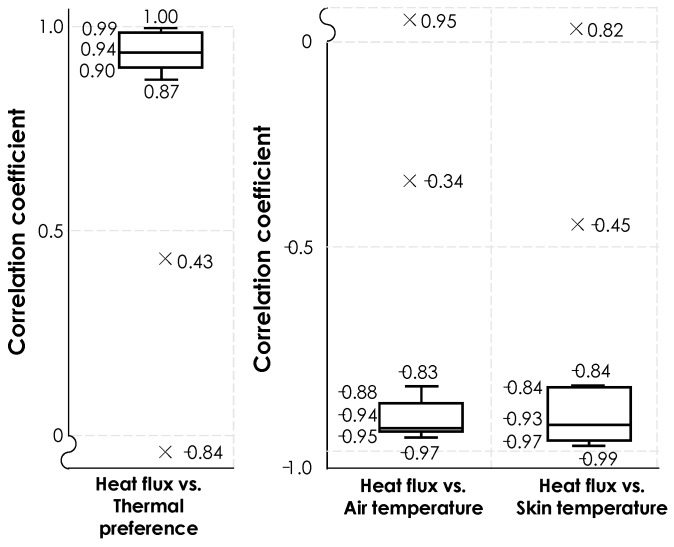
Correlation coefficients of 14 human subjects between heat flux and (1) thermal preference, (2) air temperature, and (3) skin temperature; these are the results for measurements on the facial skin.

Figure 8 presents the correlation coefficient values from correlation analyses among different parameters for the 14 participants in the first round of experiments, during which the sensor was attached to the facial skin. As Figure 8 illustrates, the heat exchange rates have a high correlation with thermal preference, air temperature, and skin temperature. We observed a positive correlation between heat exchange rates and thermal preferences, and a negative correlation for other variables. The median values of each correlation coefficient were 0.94, −0.94, and −0.93, respectively. In two cases, we observed outliers (plotted with cross marks in Figure 8). Heat flux data from a male participant showed irregularity, compared to other participants. Unstable contact between the skin and the sensor could potentially cause this problem. The other outlier was related to another participant, for whom the heat exchange rates started increasing in the middle of the experiment despite the increase in cheek skin temperature. This resulted in moderate correlation coefficients (i.e., 0.43 for the correlation coefficient between heat flux and thermal preference).

**Figure 9:**
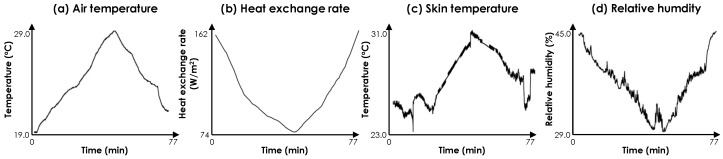
Data captured from human subject #5.

*Study of physiological variables on the wrist skin*: In Figure 9, we have presented the variations of different variables for an example participant in the second experimental study for measuring the physiological variables on the wrist skin. Similar to Figure 6, only the heat exchange rate data (Figure 9b) was post-processed through filtering for demonstration. Wrist temperature changed along with air temperature, but heat exchange rate and relative humidity changed in the opposite direction. These graphs are representative of the dataset and reflect the change of ambient temperature from low to high and to low again. Similar to the observations for the first experiment, the heat exchange rate was high at the beginning of the experiment and showed a decreasing trend as the ambient temperature increased (i.e., the first half of the experiment). During the second half of the experiment, the heat exchange rate showed an increasing trend as the temperature decreased. We did not notify the participants of the changes of the temperature during the whole experiment. During the temperature variations, 16 out of 18 participants reported positive values (requesting to have warmer environments) at the beginning of the experiment, negative values in the middle when the air temperature reached the maximum value, and once again, positive values at the end. Two subjects (one male and one female) reported only zero and positive values (requesting to have warmer environments) throughout the experiments.

**Figure 10:**
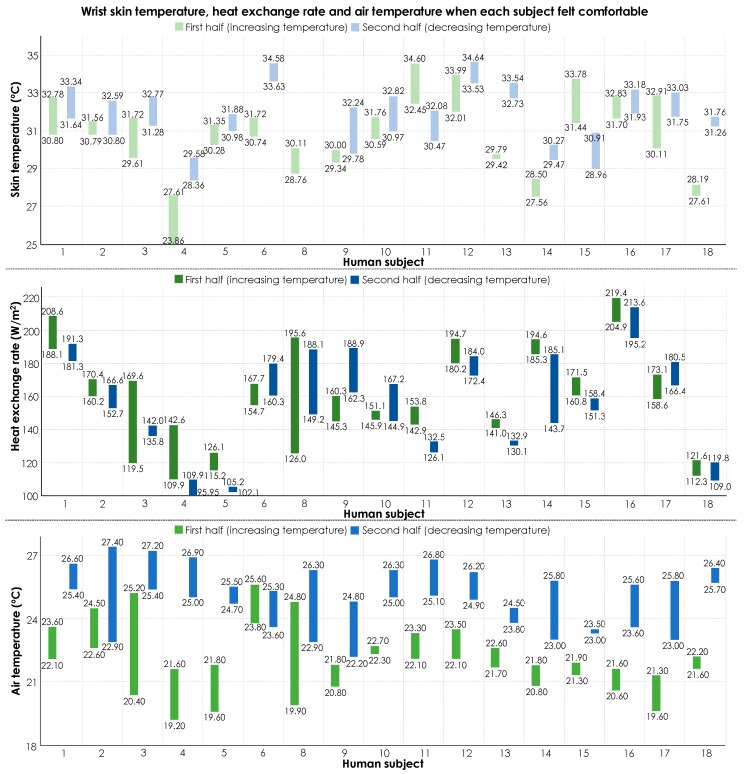
Ranges of heat exchange rate, skin temperature, and air temperature for the comfortable state under different transient temperature trends (increasing and decreasing) in the second experiment (i.e., measuring the variables on the wrist).

Similarly, in this case, in Figure 10, we present the ranges for each variable during the time that the participants expressed a comfortable state for different transient temperature trends (i.e., increasing and decreasing).

Like Figure 7, this illustration shows that individuals have different ranges of heat exchange rate and skin temperature when they feel comfortable, which emphasizes the need for personalized thermal comfort models. Another key observation is that most of the human subjects reported their zero votes (i.e., comfortable ambient conditions) while experiencing different ranges of air temperature for the increasing and decreasing transient temperature trends. For example, subject #1 felt comfortable when the air temperature was from 22.1 °C to 23.6 °C in the first half of the experiment, but, in the second half of the experiment, he was comfortable with a temperature from 25.4 °C to 26.6 °C.

The change in occupant experience at the temperature turning point could be associated to physiological or psychological factors. On the physiological side, once the skin sensors communicate the information about the drop-in temperature, the brain might interpret the experience as getting to a comfortable state considering the immediate reduction in need for thermoregulation. This observation highlights the importance of accounting for the thermal experience of occupants, which can impact the perception of thermal comfort. Furthermore, these observations shed light on the relatively low performance of thermal comfort inference models that solely rely on ambient condition parameters as features. In other words, accounting for thermal experiences could lead to a better performance in thermal comfort inference. However, since the objective of this study is to evaluate the feasibility of using heat flux as a feature for comfort-driven HVAC operations, further analyses on this observation have not been included in this study. Alternatively, by considering diverse scenarios in thermal comfort inference modeling, as presented in Table 2, we have implicitly addressed the importance of psychophysiological attributes of thermal comfort inference. As Figure 10 shows, the physiological variables are better representation of thermal comfort experiences under varied ambient conditions. In other words, the human body could potentially reflect the impact of thermal experiences in its response to environmental factors. In order to further evaluate their impact, the next section presents the results of ML modeling using different features.

**Figure 11:**
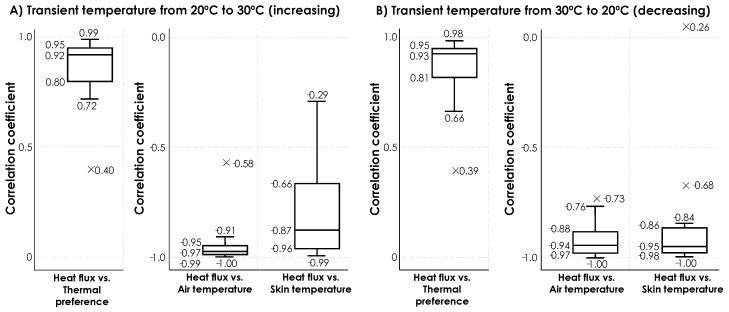
Correlation coefficients between heat exchange rate and (1) thermal preference, (2) air temperature, and (3) skin temperature for 18 participants in the second experiment for (**A**) increasing transient temperature, and (**B**) decreasing transient temperature.

**Figure 12:**
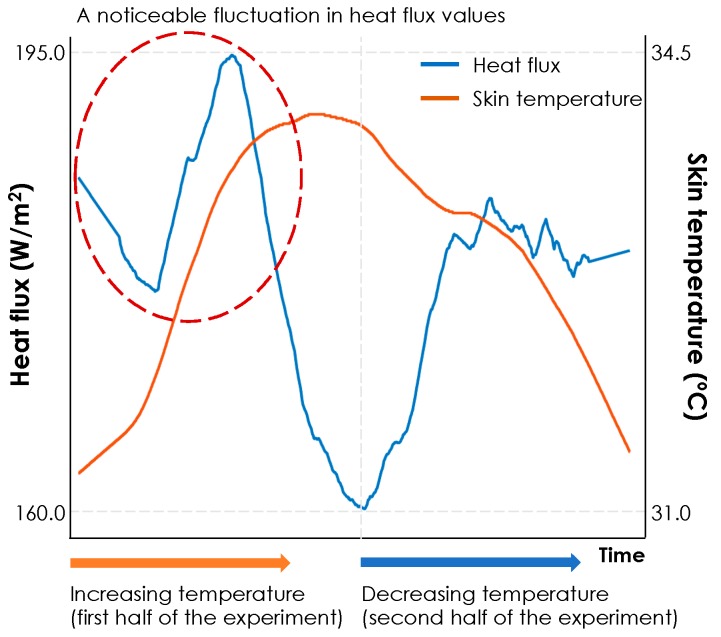
Participant #11′s heat exchange rate and skin temperature variations during the experiment.

Figure 11 shows the results of correlation analysis for the data, collected through the second experiment. We have separated the results for increasing and decreasing transient temperatures. The observed variations through the increasing transient temperatures shows a similar pattern as was observed in Figure 8**.** Heat exchange rate showed high positive correlations with thermal preferences (with a median value of 0.92), as well as high negative correlations with air temperature (with a median value of −0.97) and skin temperature (with a median value of −0.87). The correlation coefficients from the heat exchange rate and skin temperature shows a higher variance, compared to the others. For the subjects #6, 8, 9, 10, 12, and 13 we observed correlation coefficient values below −0.75 because either their skin temperature or heat exchange rate data showed fluctuations. Figure 12 illustrates an example by presenting the variation of heat exchange rate and skin temperature for human subject #11. Similarly, we also observed outliers in this experiment with different reason for the observation. For example, in this experiment, one of the participants started her thermal preference vote with one but increased it to five in the middle of the first half of experiment despite the fact that indoor temperature was gradually increasing. This observation reflects the thermal comfort sensitivity of the participants as discussed in [19,45].

### 4.2. Personalized Thermal Comfort Inference

In this section, we have presented the modeling and performance assessment for different classification algorithms. Given that we collected participants’ votes for almost same temperature ranges and stored data points at the same interval, the data set was well balanced. Therefore, we presented the performance of algorithms using accuracy as the main metric in this section. We have also presented the boxplots of F1 scores for different scenarios in the Appendix A. To this end, as described earlier in the text, we have considered different scenarios of testing and training.

**Figure 13:**
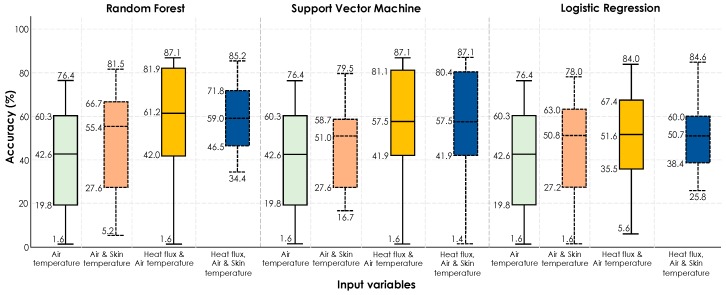
Accuracy boxplots for personal thermal comfort learning of 18 participants using three machine-learning algorithms (training on the data from the *first half of the experiment* | testing on the data from the *second half of the experiment*).

*Scenario #1 (training on first half of the experiment | testing on second half of the experiment)*: Figure 13 shows the boxplots of the accuracies for 18 human subjects obtained from each algorithm.

In general, the performance of comfort inference is lower, compared to the previous studies. When air temperature was used as the sole feature, a median accuracy of 42.6% was observed across all the models. However, when physiological attributes such as skin temperature and heat exchange rate were added, the performance improved. Nonetheless, the overall accuracies are relatively low. The main reason for the observed low accuracies is the impact of increasing and decreasing transient temperatures and the thermal comfort memory that they create. The immediate change in thermal conditions causes the comfort memory to affect the experience of participants. This is an important observation when it comes to real-world implementation of personal thermal comfort learning. Nonetheless, in all models, the heat flux feature has resulted in improved performance, even compared to the cases when skin temperature has been used. Random forest had the best performance in general as also reported in similar previous studies.

**Figure 14:**
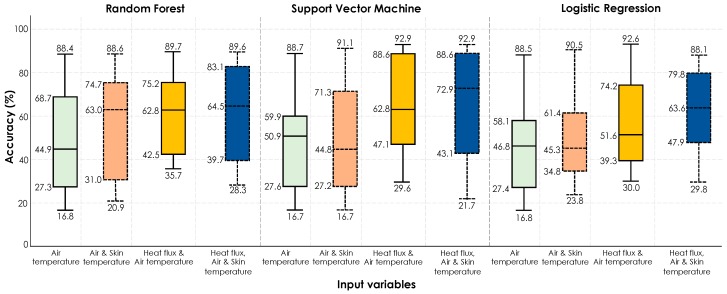
Accuracy boxplots for personal thermal comfort learning of 18 participants using three machine-learning algorithms (training on the data from the *second half of the experiment* | testing on the data from the *first half of the experiment*).

*Scenario #2 (training on the second half of the experiment | testing on the first half of the experiment)*: As it is shown in Figure 14, when the training was conducted using the data from the second half of the experiment and testing on the data from the first half of the experiment, the same conclusion on the impact of different features could be drawn in this case as well. When air temperature is used as the sole feature the algorithms manifest a poor performance. Adding the heat flux feature to the algorithms played an important role in boosting the performance, which demonstrates the efficacy of using heat flux sensors as a potential contender for HITL operation of HVAC systems. The slight improved performance in the second scenario is an interesting observation that could be associated with the fact that the data from the second half of the experiments cover the nature of participants’ experience in the first half, however, this observation is not true for the first scenario.

**Figure 15:**
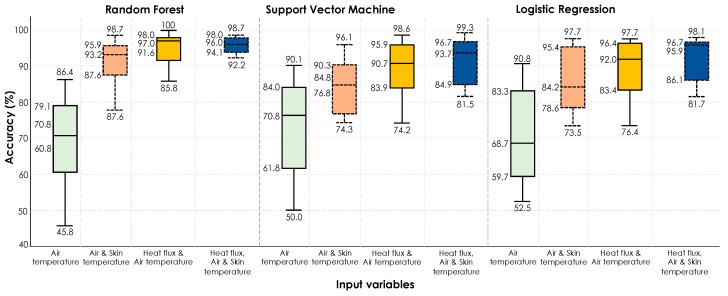
Accuracy boxplots for personal thermal comfort learning of 18 participants using three machine-learning algorithms (cross validation on all the data from the entire data set).

*Scenario #3 (cross validation on all the data points combined)*: In this scenario, we have simulated the impact of a more comprehensive training in which training and testing will be conducted on the data from both experiences. Figure 15 shows accuracy boxplots for personal thermal comfort learning of 18 participants using all the data through cross validation. As shown before, a general trend is the increase in performance by including physiological features as input parameters to the algorithms. Specifically, by using RF algorithm, which has shown the best overall performance, the median accuracy was 70.8% with air temperature as the sole feature, but it increased to 93.2% with addition of skin temperature and 97.0% with the addition of heat flux, respectively. Therefore, the results show an improved performance by using the heat exchange rate values compared to the use of commonly used skin temperature. Looking at the performance of three models, when skin temperature and heat flux were used simultaneously in addition to ambient temperature, the best performance was observed although the boost from heat exchange rate appears to be more effective.

## 5. Conclusions

In this study, by adopting a methodology comprised of experimental data collection and data analytics including machine learning modeling, we have sought to explore the feasibility and efficacy of heat flux sensors in inferring personal thermal comfort preferences toward human-in-the-loop comfort-driven HVAC operations. Given that facial and wrist areas are often exposed in indoor environments and have been used in thermal comfort modeling studies (that utilize computer-vision and wearable sensing technologies), we used the heat flux sensors on participants’ face and wrist skin to measure the variations of heat exchange rate and skin temperature under gradual transient temperature variations. Correlation between thermophysiological responses, ambient environment, human-environment heat exchange, and occupant thermal preferences were investigated. Heat exchange rate had high positive correlations with thermal preferences (a median value of 0.92 and 0.94), as well as high negative correlations with air temperature (a median value of −0.94 and −0.97) and skin temperature (a median value of −0.87 and −0.95). Furthermore, we investigated the performance of personal thermal comfort inference using classification algorithms and observed a median of 97.0% accuracy when using heat exchange rate and ambient temperature as features. The use of heat exchange rate from skin resulted in a better performance in inferring the thermal comfort preference of participants compared to the case of using skin temperature and ambient temperature as features. Therefore, the adoption of heat flux sensing was demonstrated to be promising for integration into the comfort-driven human-in-the-loop HVAC operation.

During the experiments, we observed one sensor-related limitation, namely, the need for stable contact between the sensor and the skin surface. Given the controlled nature of our experiments, this limitation did not cause any hurdles in our data processing. Nevertheless, the application of this sensing modality for field experiments further precautions are required to ensure the quality of the data. In an envisioned integration of the sensing system into wearable devices, such as prototypes for field experiments or smartwatches, in addition to design of the band for reliable skin contact, algorithms could be developed to monitor the level of contact between the skin and sensor for reliable data collection. The design of post-processing algorithms should therefore be in concert with the reliable contact time and the quality of the data that is obtained from the sensor. Furthermore, our experimental studies were performed under transient temperature conditions to investigate the sensitivity and applicability. Further feasibility studies are needed to provide an insight on the performance of the system in field studies, where the change in thermal condition and use of the sensors are less controlled.

Another practical limitation of studies on machine learning for thermal comfort inference is the provision of labeled data for training the algorithms. In the context of human thermal comfort, the personal labels are subjective votes from individuals. Studies have shown that people tend to have a low consistency in reporting similar thermal sensation votes under similar ambient thermal conditions. This is a factor that has triggered the use of human physiological response for a better association between what the body experiences compared to ambient condition measurements. To this end, although in this study, we focused on investigating the effect of skin heat exchange rate and temperature, exploring the integration of other physiological features such as heart rate and skin conductivity is an important step in future research directions. Furthermore, other environmental factors such as radiant heat (e.g., from natural or artificial light exposure), as well as room physical characteristics, such as insolation could affect the user experience. Accordingly, investigating additional physiological features, wearable device design and prototyping, the assessment of the proposed wearable technology in a field study, developing the algorithms for reliable data collection in active field studies, the impact of more complex environmental factors, such as radiant heat, and the use of the technology on assessing the required acclimation time toward the best performance of physiological sensing systems are among the future directions of this research. Furthermore, the use of more advanced machine-learning algorithms (e.g., deep learning) could be investigated to gauge its impact in terms of both automatic feature extraction and performance improvement.

## Figures and Tables

**Table 1:** Details of experimental procedures for this study.

	First Experimental Scenario	Second Experimental Scenario
Objective	Investigating the correlation between ambient environment, skin temperature, and heat exchange rate	(1) Investigating the correlation between ambient environment, skin temperature, and heat exchange rate and (2) modeling for thermal comfort inference
Measurement area	Cheek	Wrist
Number of human subjects	14 (Male: 7, Female: 7)	18 (Male: 12, Female: 6)
Temperature setup	A transient temperature variation from 20 to 30 °C	Transient temperature conditions (1) from 20 to 30 °C and (2) from 30 to 20 °C
Duration	50–60 min	100–120 min

**Table 2:** Diverse training and testing scenarios for thermal comfort inference.

Scenario	Training Dataset	Test Dataset
1	First half of the experiment (increasing temperature)	Second half of the experiment (decreasing temperature)
2	Second half of the experiment (decreasing temperature)	First half of the experiment (increasing temperature)
3	All data (Cross Validation)	All data (Cross Validation)

**Figure A1:**
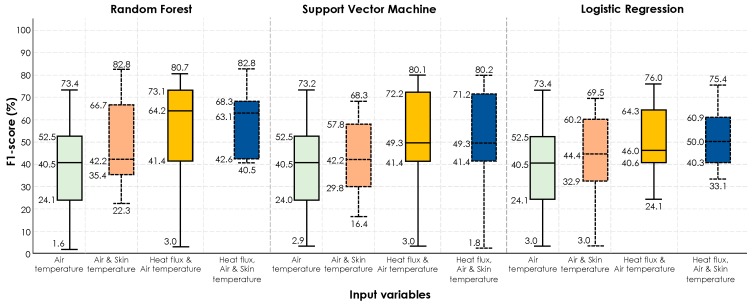
F1-score corresponding to Figure 13.

**Figure A2:**
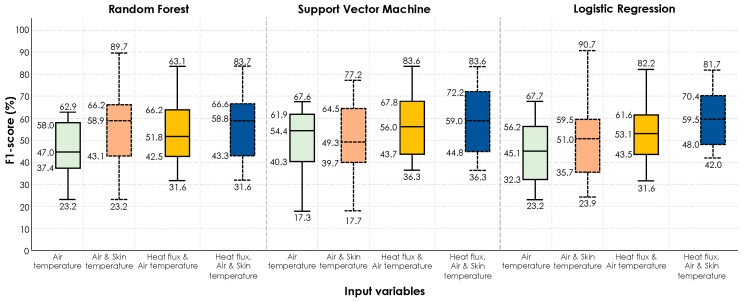
F1-score corresponding to Figure 14.

**Figure A3:**
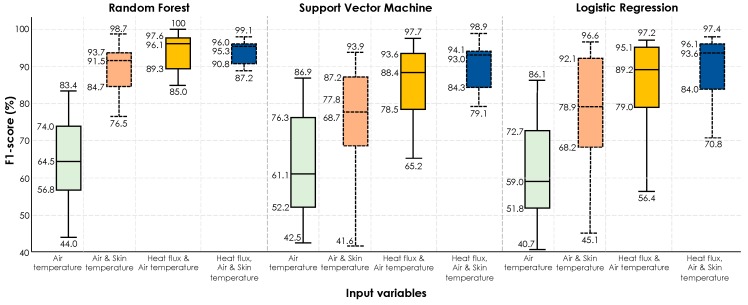
F1-score corresponding to Figure 15.

## Appendix A

The following figures present the F1 scores obtained for each scenario. Figure A1, Figure A2 and Figure A3 correspond to Figure 13, Figure 14 and Figure 15, respectively. We used boxplots to present the general trends and as it is shown, the trends are similar to those observed when using accuracy as the performance metric.

## References

[1] Jazizadeh F., Ghahramani A., Becerik-Gerber B., Kichkaylo T., Orosz M. (2014). User-led decentralized thermal comfort driven HVAC operations for improved efficiency in office buildings. Energy Build..

[2] Ghahramani A., Jazizadeh F., Becerik-Gerber B. (2014). A knowledge based approach for selecting energy-aware and comfort-driven HVAC temperature set points. Energy Build..

[3] Jazizadeh F., Ghahramani A., Becerik-Gerber B., Orosz M.T.K. Personalized Thermal Comfort Driven Control in HVAC Operated Office Buildings. Proceedings of the ASCE International Workshop on Computing in Civil Engineering (IWCCE) Conference.

[4] ASHRAE (2017). Thermal Environmental Conditions for Human Occupancy.

[5] Humphreys M.A., Hancock M. (2007). Do people like to feel ‘neutral’? Exploring the variation of the desired thermal sensation on the ASHRAE scale. Energy Build..

[6] Hoof J.J. (2008). Forty years of Fanger’s model of thermal comfort: Comfort for all?. Indoor Air.

[7] ASHRAE, SI (2017). 2017 ASHRAE® Handbook—Fundamentals.

[8] Huizenga C., Abbaszadeh S., Zagreus L., Arens E.A. (2006). Air Quality and Thermal Comfort in Office Buildings: Results of a Large Indoor Environmental Quality Survey. Proc. Healthy Build..

[9] Karjalainen S., Koistinen O. (2007). User problems with individual temperature control in offices. Build. Environ..

[10] Karjalainen S. (2009). Thermal comfort and use of thermostats in Finnish homes and offices. Build. Environ..

[11] Huizenga C., Laeser K., Arens E. (2002). A web-based occupant satisfaction survey for benchmarking building quality. Indoor Air.

[12] Cahill J., Portales R., McLoughin S., Nagan N., Henrichs B., Wetherall S. (2019). IoT/Sensor-Based Infrastructures Promoting a Sense of Home, Independent Living, Comfort and Wellness. Sensors.

[13] Salamone F., Belussi L., Currò C., Danza L., Ghellere M., Guazzi G., Lenzi B., Megale V., Meroni I. (2018). Integrated Method for Personal Thermal Comfort Assessment and Optimization through Users’ Feedback, IoT and Machine Learning: A Case Study. Sensors.

[14] Jung W., Jazizadeh F. (2019). Human-in-the-loop HVAC operations: A quantitative review on occupancy, comfort, and energy-efficiency dimensions. Appl. Energy.

[15] Jazizadeh F., Kavulya G., Klein L., Becerik-Gerber B. (2011). Continuous Sensing of Occupant Perception of Indoor Ambient Factors. Comput. Civ. Eng..

[16] Daum D., Haldi F., Morel N. (2011). A personalized measure of thermal comfort for building controls. Build. Environ..

[17] Li D., Menassa C.C., Kamat V.R. A Personalized HVAC Control Smartphone Application Framework for Improved Human Health and Well-Being. Proceedings of the Computing in Civil Engineering.

[18] Li D., Menassa C.C., Kamat V.R. (2017). Personalized human comfort in indoor building environments under diverse conditioning modes. Build. Environ..

[19] Jung W., Jazizadeh F. (2018). Multi-Occupancy Indoor Condition Optimization in consideration of Thermal Sensitivity. Advanced Computing Strategies for Engineering.

[20] Jazizadeh F., Ghahramani A., Becerik-Gerber B., Kichkaylo T., Orosz M. (2014). Human-Building Interaction Framework for Personalized Thermal Comfort-Driven Systems in Office Buildings. J. Comput. Civ. Eng..

[21] Kim J., Zhou Y., Schiavon S., Raftery P., Brager G. (2018). Personal comfort models: Predicting individuals’ thermal preference using occupant heating and cooling behavior and machine learning. Build. Environ..

[22] Ghahramani A., Tang C., Becerik-Gerber B. (2015). An online learning approach for quantifying personalized thermal comfort via adaptive stochastic modeling. Build. Environ..

[23] Li D., Menassa C.C., Kamat V.R. (2018). Non-intrusive interpretation of human thermal comfort through analysis of facial infrared thermography. Energy Build..

[24] Choi J.-H., Loftness V. (2012). Investigation of human body skin temperatures as a bio-signal to indicate overall thermal sensations. Build. Environ..

[25] Choi J.H. (2010). CoBi: Bio-Sensing Building Mechanical System Controls for Sustainably Enhancing Individual Thermal Comfort. Ph.D. Thesis.

[26] Ranjan J., Scott J. ThermalSense: Determining Dynamics Thermal Comfort Preferences Using Thermographic Imaging. Proceedings of the 2016 ACM International Joint Conference on Pervasive and Ubiquitous Computing.

[27] Yi B., Choi J.H. Facial Skin Temperature as a Proactive Variable in a Building Thermal Comfort Control System. Proceedings of the First International Symposium on Sustainable Human-Building Ecosystems.

[28] Jung W., Jazizadeh F. Towards Integration of Doppler Radar Sensors into Personalized Thermoregulation-Based Control of HVAC. Proceedings of the 4th ACM Conference on Systems for Energy-Efficient Built Environment.

[29] Jung W., Jazizadeh F. (2018). Vision-based thermal comfort quantification for HVAC control. Build. Environ..

[30] Jazizadeh F., Jung W. (2018). Personalized thermal comfort inference using RGB video images for distributed HVAC control. Appl. Energy.

[31] Jazizadeh F., Kavulya G., Kwak J., Becerik-Gerber B., Tambe M., Wood W. Human-building interaction for energy conservation in office buildings. Proceedings of the Construction Research Congress.

[32] Jung W., Jazizadeh F. Non-Intrusive Detection of Respiration for Smart Control of HVAC System. Proceedings of the Computing in Civil Engineering.

[33] Jung W., Jazizadeh F. Towards Non-intrusive Metabolic Rate Evaluation for HVAC control. Proceedings of the 17th International Conference on Computing in Civil and Building Engineering.

[34] Jazizadeh F., Pradeep S. Can computers visually quantify human thermal comfort? Short Paper. Proceedings of the 3rd ACM International Conference on Systems for Energy-Efficient Built Environments.

[35] Sim S.Y., Koh M.J., Joo K.M., Noh S., Park S., Kim Y.H., Park K.S. (2016). Estimation of Thermal Sensation Based on Wrist Skin Temperatures. Sensors.

[36] Qi H., Guo Z., Chen X., Shen Z., Wang Z.J. (2017). Video-based human heart rate measurement using joint blind source separation. Biomed. Signal Process. Control..

[37] Fanger P.O. (1970). Thermal Comfort. Analysis and Applications in Environmental Engineering.

[38] Choi J.H., Loftness V., Lee D.W. (2012). Investigation of the possibility of the use of heart rate as a human factor for thermal sensation models. Build. Environ..

[39] Liu W., Lian Z., Liu Y. (2008). Heart rate variability at different thermal comfort levels. Graefe’s Arch. Clin. Exp. Ophthalmol..

[40] Wang X., Li D., Menassa C.C., Kamat V.R. (2019). Investigating the effect of indoor thermal environment on occupants’ mental workload and task performance using electroencephalogram. Build. Environ..

[41] Jebelli H., Hwang S., Lee S. (2018). EEG-based workers’ stress recognition at construction sites. Autom. Constr..

[42] Jebelli H., Lee S. (2019). Feasibility of Wearable Electromyography (EMG) to Assess Construction Workers’ Muscle Fatigue. Advances in Informatics and Computing in Civil and Construction Engineering.

[43] Jung W., Jazizadeh F. (2019). Comparative assessment of HVAC control strategies using personal thermal comfort and sensitivity models. Build. Environ..

[44] Abedi M., Jazizadeh F., Huang B., Battaglia F. (2018). Smart HVAC Systems—Adjustable Airflow Direction. Advanced Computing Strategies for Engineering.

[45] Klein L., Kavulya G., Jazizadeh F., Kwak J.Y., Becerik-Gerber B., Varakantham P., Tambe M. Towards optimization of building energy and occupant comfort using multi-agent simulation. Proceedings of the 28th International Symposium on Automation and Robotics in Construction (ISARC).

[46] Kwak J.Y., Varakantham P., Maheswaran R., Tambe M., Jazizadeh F., Kavulya G., Klein L., Becerik-Gerber B., Hayes T., Wood W. SAVES: A sustainable multiagent application to conserve building energy considering occupants. Proceedings of the 11th International Conference on Autonomous Agents and Multiagent Systems-Volume 1 (AAMAS).

[47] Kwak J.Y., Varakantham P., Tambe M., Klein L., Jazizadeh F., Kavulya G., Gerber B.B., Gerber D.J. Towards Optimal Planning for Distributed Coordination under Uncertainty in Energy Domains. Proceedings of the Workshop on Agent Technologies for Energy Systems (ATES).

[48] FluxTeq, FluxTeq Heat Flux Sensor. http://www.fluxteq.com/.

[49] ASTM E2684-17 (2017). Standard Test Method for Measuring Heat Flux Using Surface-Mounted One-Dimensional Flat Gages.

[50] Kreith F. (2005). The CRC Handbook of Mechanical Engineering, Second Edition Heat and Mass Transfer. The CRC Handbook of Mechanical Engineering.

[51] Jung W., Chan M., Jazizadeh F., Diller T.E. Feasibility Assessment of Heat Flux Sensors for Human-in-the-Loop HVAC Operations. Proceedings of the ASCE International Conference on Computing in Civil Engineering 2019.

[52] Dabiri S., Jazizadeh F. Exploring video based thermal perception identification. Proceedings of the 16th International Conference on Computing in Civil and Building Engineering.

[53] Jazizadeh F., Marin F.M., Becerik-Gerber B. (2013). A thermal preference scale for personalized comfort profile identification via participatory sensing. Build. Environ..

[54] Shenoy S.K., Diller T.E. (2018). Heat flux measurements from a human forearm under natural convection and isothermal jets. Int. J. Heat Mass Transf..

[55] Schafer R.W. (2011). What Is a Savitzky-Golay Filter?. IEEE Signal Process. Mag..

